# Empowering adolescents living with perinatally-acquired HIV: tailored CD4+ count assessment for optimized care, the EDCTP READY-study

**DOI:** 10.3389/fmed.2024.1457501

**Published:** 2024-09-20

**Authors:** Willy Le Roi Togna Pabo, Aurelie Minelle Kengni Ngueko, Alex Durand Nka, Maria Mercedes Santoro, Yagai Bouba, Collins Ambe Chenwi, Ezéchiel Ngoufack Jagni Semengue, Désiré Takou, Georges Teto, Beatrice Dambaya, Raymond Babila Nyasa, Michel Carlos Tommo Tchouaket, Grace Angong Beloumou, Sandrine Claire Djupsa Ndjeyep, Aude Christelle Ka’e, Tatiana Anim Keng Tekoh, Derrick Tambe Ayuk Ngwese, Naomi-Karell Etame, Rachel Audrey Nayang Mundo, Rachel Simo Kamgaing, Samuel Martin Sosso, Roland Ndip Ndip, Vittorio Colizzi, Francesca Cecchereni-Silberstein, Alexis Ndjolo, Joseph Fokam

**Affiliations:** ^1^Chantal BIYA International Reference Centre for Research on HIV/AIDS Prevention and Management (CIRCB), Yaoundé, Cameroon; ^2^Faculty of Science, University of Buea, Buea, Cameroon; ^3^Faculty of Medicine and Health Sciences, University of Antwerp, Antwerp, Belgium; ^4^University of Rome “Tor Vergata”, Rome, Italy; ^5^School of Health Sciences, Catholic University of Central Africa, Yaoundé, Cameroon; ^6^Evangelical University of Cameroon, Bandjoun, Cameroon; ^7^Faculty of Medicine and Biomedical Sciences University of Yaoundé I, Yaoundé, Cameroon; ^8^National HIV Drug Resistance Working Group, Ministry of Public Health, Yaoundé, Cameroon; ^9^Faculty of Health Science, University of Buea, Buea, Cameroon

**Keywords:** HIV, immune monitoring, CD4 cell count, adolescents, Cameroon

## Abstract

**Background:**

The elevated rate of AIDS-related mortality in Sub-Saharan Africa among adolescents living with HIV (ALHIV) is influenced by various factors, notably immunosuppression, within a framework of limited therapeutic alternatives. We aimed to enhance the management of pediatric HIV by assessing the immune response and associated factors in perinatally-infected ALHIV on antiretroviral therapy (ART) in Cameroon.

**Methods:**

A cohort study was conducted from 2018–2020 among 271 ART-experienced ALHIV in Cameroon. Sociodemographic data, immunological (CD4), and virological (plasma viral load, PVL) responses were measured at enrolment (T0), 6-months (T1), and 12-months (T2) using PIMA CD4 (Abbott/Pantech (Pty) Ltd) and Abbott Applied Biosystem platform (Real-Time PCR m2000RT) respectively. Immunological failure (IF) was defined as absolute CD4 < 250 cells/mm^3^, and Virological failure (VF) as PVL ≥ 1,000 copies/ml. A linear mixed-effects model with R version 4.4.1 was used to estimate both fixed and random effects, with significance set at *p* < 0.05.

**Results:**

Of the 271 perinatally-infected ALHIV enrolled over three phases, females were predominant (55.7, 55.1, and 56.0%); median age was 14 (IQR: 12–17); majority of the participants were followed-up in urban areas (77.5, 74.5, and 78.6%); and the age distribution favored older adolescents (48.7, 61.2, and 58.5%). Most participants achieved clinical success (93.1, 89.7, 88.9%), predominantly on first-line ART (80.8, 66.2, and 53.0%), with good adherence (64.2, 58.9, and 64.5%). Most participants had secondary education (67.2, 70.1, and 67.5%). Median CD4+ counts fluctuated overtime, with values of 563 (IQR: 249.0–845.0), 502 (IQR: 319.0–783.5), and 628 (IQR: 427.5–817.5), respectively. Of note, being male was linked to a reduction in CD4+ count compared to females, [−200.63 (−379.32 to −21.95), *p* = 0.028]. Similarly, late adolescence was associated with lower CD4+ counts compared to early adolescence, [−181.08 (−301.08 to −61.09), *p* = 0.003]. Moreover, participants experiencing VF showed significantly lower CD4+ counts compared to those with undetectable viral loads, [−353.08 (−465.81 to −240.36), *p* < 0.001]. Additionally, there was a marginally significant interaction between male gender and secondary educational level, [209.78 (−6.94–426.51), *p* = 0.058].

**Conclusion:**

Among perinatally-infected ALHIV, age, gender, educational level, and virological status are key factors influencing their immune health and treatment outcomes. Prioritizing targeted interventions and close monitoring within these subgroups is crucial for optimal management, employing holistic care strategies that consider not only medical interventions but also psychosocial support and education.

## Background

Sub-Saharan Africa (SSA) bears the highest global burden of HIV, encompassing 54.6% of the 38.0 million infections and 43.7% of the 690,000 [500,000–970,000] AIDS-related deaths (1). Adolescents, constituting a vulnerable demographic, contribute to approximately 49.3% of new infections in this region (1). Cameroon, one of the 15 countries severely affected by HIV among adolescents, contributes 2% to the global HIV prevalence in this population (2). Despite a decline in AIDS-related mortality attributed to expanded antiretroviral therapy (ART), adolescent mortality has risen by approximately 50%, primarily due to opportunistic infections (3). Adolescents with HIV pose a significant health concern with limited routine management data, especially in SSA where surveillance strategies remain suboptimal without robust clinical evidence (4–6).

Historically, the CD4 cell count served as the primary marker for immune suppression assessment and ART initiation timing in HIV/AIDS patients (7, 8). However, the 2015 World Health Organization (WHO) recommendation advocates for ART initiation irrespective of CD4 count, symptoms, or clinical conditions (9). Some subsequent studies propose reduced or discontinued CD4 monitoring post-ART initiation when viral load tests are available and show viral load suppression (10, 11). The paradigm shifts toward treatment for all, viral load implementation, and suggestions to curtail CD4 testing post-treatment initiation prompt a re-evaluation of the significance of CD4 testing. Intriguingly, the 2017 WHO guideline strongly advocates for a comprehensive care package, including close monitoring of viral load and CD4, for patients with advanced HIV disease (CD4 count below 200 cells/mm^3^) (12).

Beyond the CD4 T cell count, viral load significantly influences treatment outcomes (13). Elevated baseline viral loads indicate heightened HIV replication and increased immune system strain, possibly necessitating more aggressive treatment strategies for viral suppression (14). It is crucial to acknowledge that while baseline immune status strongly predicts treatment outcomes, it is not the sole determinant (14). Factors like treatment adherence, drug resistance, and comorbidities also impact treatment response. Hence, a holistic approach considering multiple factors becomes imperative to optimize treatment outcomes for ALHIV (15). The immunological status of ALHIV undergoing ART plays a pivotal role in predicting treatment outcomes. Elevated baseline CD4+ T cell counts are linked to more favorable treatment responses and enhanced long-term results. Conversely, diminished CD4+ T cell counts and heightened viral loads at baseline signify an increased risk of complications, necessitating more rigorous monitoring and intervention (16). However, it is imperative to take into account other factors influencing treatment response to formulate a comprehensive approach for managing HIV infection in this adolescent population. Given the paucity of information on immuno-virological monitoring in pediatric populations, especially in developing countries, this study aimed to evaluate the prevalence of adolescents living with HIV (ALHIV) requiring immune monitoring and investigate the association between immunological markers and virological failure (VF) in this population.

## Methods

### Study design and setting

A prospective cohort study was carried out from 2018 to 2020 among 271 ALHIV undergoing ART at designated health facilities within the “Resistance Evolution among Adolescents in Yaoundé and its surroundings” (READY-study) in the Centre region of Cameroon. Participants were enrolled through consecutive sampling methods, and follow-ups occurred at enrolment (T0), 6 months (T1), and 12 months (T2).

### Description of study sites

The study sites were selected from both urban and rural settings within the Centre region of Cameroon, and were classified as follows:

*Urban settings*: Two health facilities specialized in pediatric care, located in Yaoundé: the Mother–Child Centre of the Chantal Biya Foundation (MCC-FCB) and the National Social Welfare Hospital (NSWH);

*Rural settings*: Two health facilities situated 30–50 km from the urban area: the Mbalmayo District Hospital (HDMB) in Mbalmayo and the Mfou District Hospital (HDMF) in Mfou. Additionally, the Nkomo Integrated Health Centre (NIHC) and the Bikop Catholic Health Centre (BCHC) served as satellite sites in the Centre region of Cameroon.

### Study site selection criteria and sampling method

The site selection for the study was based on several key criteria. Facilities were required to have at least three years of experience in managing pediatric ART, ensuring a robust background in treatment. Additionally, clinics needed to provide both first-and second-line ART regimens at their pharmacies and adhere to national ART guidelines, demonstrating compliance with established national standards. The availability of ART registers, patient medical files, or a database was also necessary to facilitate comprehensive data management. Each selected site had to cater to a minimum of 15 adolescents on ART to ensure a sufficient sample size. Lastly, to ensure a diverse representation, facilities were chosen from both urban and rural areas. An exhaustive and non-randomized sampling method was used for participant enrolment, following eligibility criteria.

### Patient characteristics

Participants were enrolled from the selected study sites. Inclusion criteria encompassed adolescents perinatally infected with HIV (APHI) within the age range of 10 to 19 years, possessing documented infection routes, and undergoing a standard antiretroviral therapy (ART) regimen based on either a first-line reverse transcriptase inhibitor (RTI) or a second-line regimen comprising a Ritonavir-boosted protease inhibitor (PI/r) for a minimum of 6 months. Additionally, compliance with the criteria involved providing written assent and securing informed consent from their legal guardian(s). Non-inclusion criteria involved individuals not formally enrolled in any ART monitoring system, reported as ART-naïve, on a drug regimen not aligned with national guidelines, or experiencing treatment interruption. Exclusion criteria encompassed participants who voluntarily withdrew from the study or transferred out of a study site before reaching mid-or endpoint evaluations.

### Laboratory procedures

Following enrolment, ten mL of whole blood were collected from each participant and transported on icepacks within 6 h to the virology laboratory of the Chantal Biya International Reference Centre (CIRCB) in Yaounde, Cameroon. A 200 μL whole blood aliquot for CD4 enumeration was obtained. Subsequently, plasma aliquots for Viral Load (PVL) and HIV genotypic resistance testing (GRT) were extracted after centrifugation at 1,600 rpm for 10 min and then stored at −80°C for further analyses. CD4 cell count was conducted using the Pima CD4 (Abbott/Pantech (Pty) Ltd., Westville, South Africa) automatic test, following the manufacturer’s instructions (17). The Pima test consists of a disposable Pima test cartridge, containing dried reagents, and the Pima Analyzer. A low sample volume of approximately 25 microliters (μL) of whole blood is collected into the test cartridge, which is then capped. The Pima test cartridge is inserted into the Pima Analyzer and the sample sealed within the cartridge is processed. During the course of test processing, data is recorded, analyzed and interpreted using software embedded in the Pima Analyzer. Upon completion of the test the cartridge is removed from the Pima Analyzer and a test result is displayed.

PVL measurement utilized the Abbott Applied Biosystem platform (Real-Time PCR AB m2000RT), adhering to the manufacturer’s instructions (Abbott Laboratories, USA) (18), with a detection threshold of 40 copies/mL (lower) and 10,000,000 copies/mL (upper). Viral RNA was extracted from 600 μL Plasma samples and carefully prepared (remove inhibitors that can interfere with the PCR reaction) for analysis. After then, 25 μL of elution buffer and 65 μL of nuclease free water was add on extracted RNA; 50 μL of eluted RNA was homogenised with Master mix (50 μL per reaction) containing reagents, primers, probes, and enzymes. The m2000RT system analyses the real-time fluorescence data and calculates the viral load in copies/mL of plasma.

To enhance polymerase chain reaction (PCR) amplification sensitivity, HIV-1 RNA extraction was performed from 1,000 μL of plasma aliquots, involving an initial 2-h refrigerated centrifugation step at 14,000 rpm to concentrate viral RNA (19). Manual extraction of HIV-1 RNA was carried out from 140 μL of plasma using the QIAGEN protocol (QIAamp® DNA Minikit; QIAGEN, Courtaboeuf, France) (20) which after the Lysis step done by adding 200 μL of Lyzol, a phase separation is done by adding 200 μL of chloroform and the RNA isolated is carefully transferred to a new 1.5 mL Eppendorf which purification with ethanol at 70% and wash solutions brought with the Kit then follows. It is finally eluted with 50 μL of elution buffer (AVE Buffer) which is now ready for the RT-PCR.

### Data management and quality control

The socio-demographic, clinical and biological data of each participant was stored in a database with access restricted to authorized persons, and each participant was assigned an identification number which was used throughout the data analysis process. CIRCB is a government institute of the Ministry of Public Health, in charge of research and reference clinical monitoring of HIV-infected patients, with participation in external quality assurance programs for HIV screening/diagnosis, viral load measurements, CD4 count, as well as biochemistry and hematological analysis.^1^

### Definition of terms

Young adolescents referred to participants aged between 10–14 years, and old adolescents referred to participants aged between 15–19 years. Virological failure (VF) was defined as PVL ≥1,000 RNA copies/mL; Virological suppression (*VS*) referred to PVL between 50–999 copies/mL and undetectable viremia referred to PVL < 50 copies/mL (21); immunological failure was defined as CD4+ cell count <250 cells/mm^3^, and adequate immune response as CD4+ cell ≥250 cells/mm^3^ (22). The primary outcome for immune monitoring was defined as CD4+ cell count >250 cells/mm^3^ as recommended by WHO (23).

### Statistical analysis

Data were initially recorded in an Excel spreadsheet and subjected to a rigorous double-checking process to ensure accuracy. Subsequently, the meticulously cleaned dataset underwent analysis using SPSS and RStudio version 2023.09.1 + 494, with a predetermined statistical significance level set at *p* < 0.05. Data for continuous variables were presented as mean and standard deviation (SD) for normally distributed, or median and interquartile range (IQR) for non-normally distributed variables. Data for categorical variables were presented as frequencies and percentages, and the data were succinctly summarized using tables and figures. Chi-square and *Fisher’s* exact tests were employed to discern associations between categorical variables, as deemed appropriate. Additionally, the *Spearman* correlation and the *Post Hoc test of Tukey* were implemented to ascertain the impact of different categories within variables on the outcome. Linear regression was used to explore factors associated with immunological response in participants with complete data on the outcome and covariates. The outcome of the model was absolute CD4+ count; model selection was done by best subset selection approach with Akaike Information Criterion (AIC) (24). “The Akaike” Information Criterion (AIC) is a statistical tool used to assess the relative quality of different models by balancing model fit and complexity. It evaluates both the likelihood of the model and the number of parameters, with lower AIC values indicating a more favorable trade-off between goodness of fit and simplicity. Thus, models with lower AIC scores are generally preferred for their more efficient representation of the data. Possible clinically meaningful interactions between variables were investigated. To account for the correlation between participants in the same study site, we expanded the multivariate linear model to a linear mixed-effect model with study site as a random effect and age ranges, gender, educational level, age at HIV diagnosis, ART line, adherence, clinical stages, and viral load ranges as fixed effects. The significance of the random effect was tested by the log-likelihood ratio test. Assumptions of the final linear regression model (linearity, normality, homoscedasticity, and outliers) were examined based on graphical examination. All statistical tests in this report were performed with a complete case analysis approach.

## Results

### Sociodemographic, biological, and clinical characteristics

In total, 271 adolescents living with HIV (ALHIV) were initially enrolled during the first phase (T1), with subsequent follow-up of 263 participants at the 6-month mark (T2), and 234 ALHIV at the 12-month mark (T3). From enrolment (T1) to 12-month assessment (T3), the majority of ALHIV were followed up in urban areas, with proportions of 210 (77.5%), 196 (74.5%), and 184 (78.6%) respectively (Table 1). Regarding the age distribution, from T1 to T3, 139 (51.3%) ALHIV were aged 10–14 years, while 161 (61.2%), and 137 (58.5%) fell into the older age category, respectively (Table 1). The median age at diagnosis was 5 years (IQR: 2–10), and the median duration on treatment was 36 months (IQR: 21–81) at T1, 30 months (IQR: 10–55) at T2, and 23 months (IQR: 9–54) at T3. In terms of gender distribution, at all-time points, there were more female participants than males, 151 (55.7%), 145 (55.1%), and 131 (56.0%) from T1 – T3, respectively (Table 1). Regarding educational level, the majority of participants had secondary education across all time points. At T1, 182 (67.2%) were in secondary school, while at T2, the number increased to 184 (70.1%). At T3, 158 (67.5%) participants had secondary education (Table 1).

**Table 1:** Sociodemographic, biological, and clinical characteristics of the study population.

	Enrolment (T1)		6-months (T2)		12-months (T3)	
	Frequency, *n* = 271	Percentage, (%)	Frequency, *n* = 263	Percentage, (%)	Frequency, *n* = 234	Percentage, (%)
Site group						
Rural	61	22.5	67	25.5	50	21.4
Urban	210	77.5	196	74.5	184	78.6
Age (years)						
10–14	139	51.3	102	38.8	97	41.5
15–19	132	48.7	161	61.2	137	58.5
Age (years) [median, (IQR)]	14 (12–17)		15 (13–18)		15 (13–17)	
Age at diagnosis [Median (IQR)]	5 (2–10)					
Duration on treatment (months) [Median, (IQR)]	36 (21–81)		30 (10–55)		23 (9–54)	
Gender						
Female	151	55.7	145	55.1	131	56.0
Male	120	44.3	118	44.9	102	44.0
Educational level						
Primary	81	29.9	68	25.8	65	27.8
Secondary	182	67.2	184	70.1	158	67.5
University	2	0.7	8	3.0	5	2.1
Not in school	3	1.1	3	1.1	2	0.9
Missing	3	1.1	-	-	4	1.7
Clinical stage						
I/II	252	93.1	236	89.7	208	88.9
III/IV	15	5.5	27	10.3	21	9.0
Missing	4	1.5	-	-	5	2.1
ART line						
First	219	80.8	174	66.2	124	53.0
Second	46	17.1	80	30.4	91	38.9
Missing	6	2.2	9	3.4	19	8.1
Adherence						
Good	174	64.2	155	58.9	151	64.5
Poor	87	32.1	108	41.1	76	32.5
Missing	10	3.7	-	-	7	3.0
Median (IQR) CD4 count	563 (249.0–845.0)		502 (319.0–783.5)		628 (427.5–817.5)	
CD4 classes						
≥250	204	75.3	214	81.4	211	90.2
<250	67	24.7	49	18.6	23	9.8
Median (IQR) PVL	60 (20–25,299.50)		95.5 (20.0–14,100.0)		53.5 (20–2,537)	
PVL classes						
≥1,000	106	39.1	103	39.1	65	27.8
<1,000	161	59.4	153	58.2	169	72.2
Missing	4	1.5	7	2.7	-	-
ART regimen						
First line RTI-/INSTI-based*						
ABC + 3TC + EFV	44	16.2	40	15.2	17	7.3
ABC + 3TC + NVP	1	0.4	-	-	-	-
AZT + 3TC + DTG	-	-	-	-	1	0.4
AZT + 3TC + EFV	28	10.3	16	6.1	11	4.7
AZT + 3TC + NVP	65	24.1	51	19.4	35	15.1
D4T + 3TC + NVP	4	1.5	-	-	-	-
TDF + 3TC + EFV	74	27.3	67	25.5	65	27.8
TDF + 3TC + NVP	3	1.1	1	0.4	2	0.8
Second line PI/*r*-based**						
ABC + 3TC + ATV/*r*	5	1.8	6	2.3	6	2.6
ABC + 3TC + LPV/*r*	5	1.8	7	2.6	6	2.6
ABC + AZT + LPV/*r*	1	0.4	-	-	-	-
ABC + DDI + LPV/*r*	1	0.4	-	-	-	-
AZT + 3TC + ATV/*r*	2	0.7	3	1.1	8	3.4
AZT + 3TC + LPV/*r*	2	0.7	10	3.8	13	5.6
AZT + DDI + LPV/*r*	1	0.4	-	-	-	-
TDF + 3TC+ ATV/*r*	18	6.6	41	15.6	57	24.4
TDF + 3TC + ATV/*r* + DTG	-	-	-	-	1	0.4
TDF + 3TC + LPV/*r*	9	3.3	12	4.5	8	3.4
TDF + ABC + LPV/*r*	1	0.4	-	-	-	-
TDF + DDI + LPV/*r*	1	0.4	-	-	-	-
Missing	6	2.2	9	3.4	4	1.7

Overall, the majority of participants achieved clinical success, classified in clinical stages I/II, with 252 (93.1%), 236 (89.7%), and 208 (88.9%) participants, respectively. In terms of ART lines, the majority of participants were on the first line, with frequencies of 219 (80.8%), 174 (66.2%), and 124 (53.0%) at each respective time point (Table 1). Regarding adherence levels, the majority of participants reported good adherence, with frequencies of 174 (64.2%), 155 (58.9%), and 151 (64.5%) across the three time points. Furthermore, the median CD4+ count fluctuated over time, with values of 563 (IQR: 249.0–845.0) at T1, 502 (IQR: 319.0–783.5) at T2, and 628 (IQR: 427.5–817.5) at T3. Similarly, the median PVL also varied across time points, with values of 60 (IQR: 20–25,299.50) at T1, 95.5 (IQR: 20.0–14,100.0) at T2, and 53.5 (IQR: 20–2,537) at T3 (Table 1). The ART regimen data showed diverse combinations of first and second-line treatments, with some variations observed over the follow-up periods (Table 1).

### Immunological response and sociodemographic characteristics

**Figure 1:**
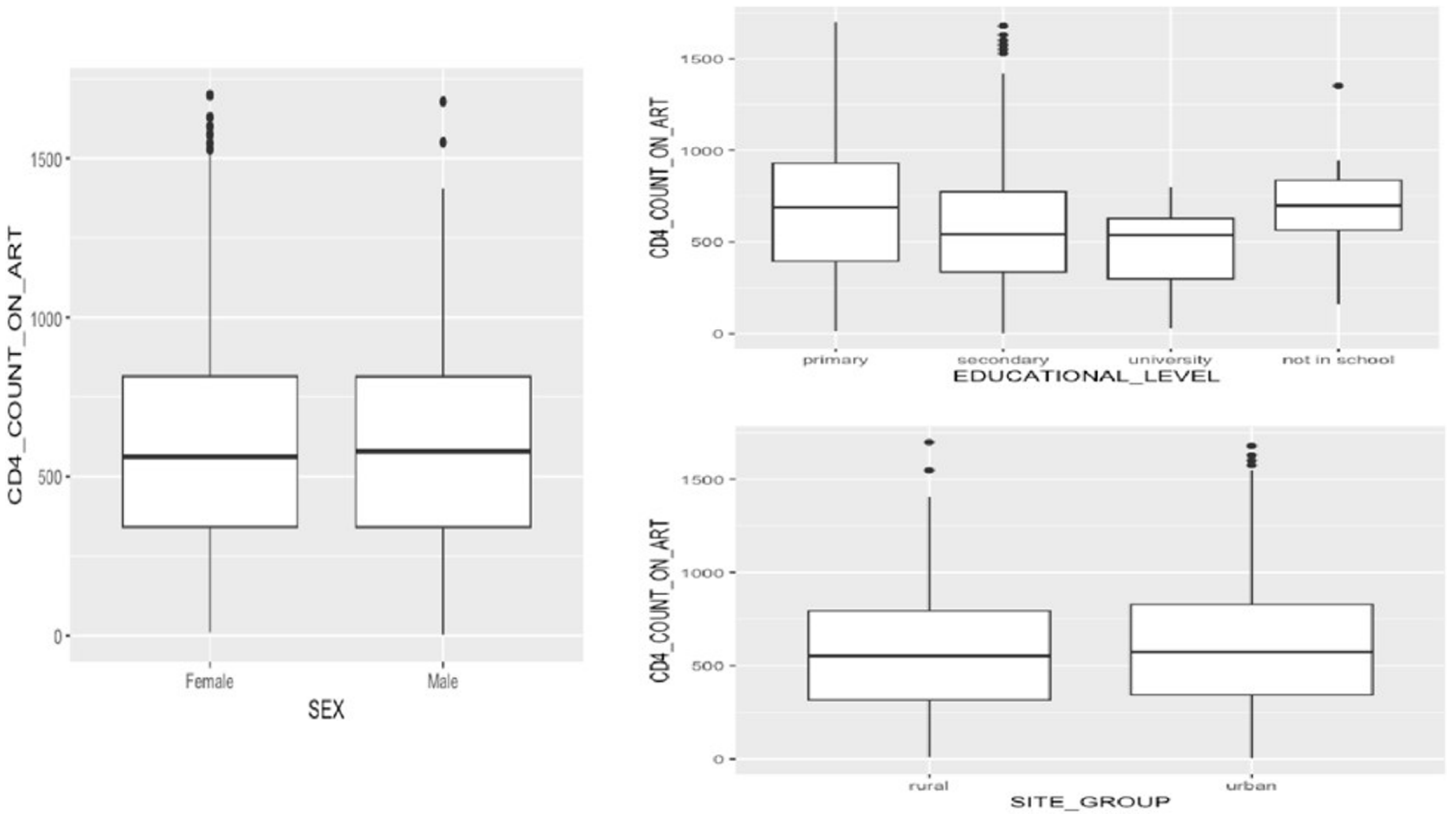
Exploratory analysis of CD4+ count dynamics in adolescents living with HIV: *impact of gender (sex), educational level, and study site characteristics*.

**Figure 2:**
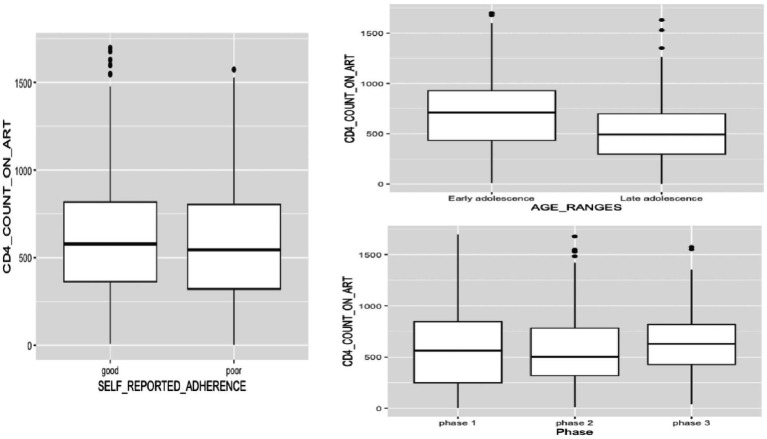
CD4+ count patterns among adolescents living with HIV: influence of self-reported adherence, adolescence age ranges, and follow-up period.

In our analysis, we observed several key findings regarding CD4+ count among ALHIV. First, we found a comparable median absolute CD4+ count between male and female adolescents (Figure 1), and this remained consistent across the study duration, with no statistically significant differences detected (*p* > 0.05) (Table 2). Additionally, comparing absolute CD4+ counts between participants followed up in urban and rural sites across different phases of the study rendered non-significant outcomes (Figure 1; Table 2). Conversely, assessing the effect of longitudinal follow-up phases on immunological responses, we observed a statistically significant difference in CD4+ count across various follow-up phases of the longitudinal study (*p* = 0.021) (Figure 2; Table 3). Regarding educational level, we observed varying median CD4+ counts, with highest values among participants with primary education, followed by secondary education, and the lowest values among participants with university education (Figure 1; Table 2).

**Table 2:** Median CD4+ count across study population characteristics.

	Enrolment (T1)			6-months (T2)			12-months (T3)		
	Median CD4+	IQR	*p*	Median CD4+	IQR	*p*	Median CD4+	IQR	*p*
Gender			0.549			0.808			0.349
Female	543.5	247.5–851.3		532.0	323.8–805.3		609.0	425.3–795.0	
Male	582.0	255.5–816.5		492.0	316.0–753.0		679.0	430.0–855.0	
Study site									
Rural	452.0	232.0–822.0		482.0	197.5–806.0		679.0	526.0–755.0	
Urban	578.0	251.5–847.3	0.296	528.5	340.0–771.8	0.472	611.5	417.8–853.3	0.849
Age group									
10–14	691.5	388.3–919.3	<0.0001	635.0	405.5–902.0	0.0005	771.0	578.0–939.0	<0.0001
15–19	419.0	213.0–688.0		464.0	292.3–670.0		543.0	379.5–717.0	
Educational level									
Primary	706.5	300.3–915.5		492.0	287.0–899.0		755.0	528–935.0	0.264
Secondary	500.0	248.5–760.0		502.0	330.5–765.0		575.0	417.8–792.8	
University	289.5	195.8–383.3		558.5	300.5–621.5		584.0	326.0–796.0	
No school	946	764.5–1148.5	0.057	544.0	351.5–621.0	0.013	729.0	-	
ART line									
First	564.0		0.876	557.0	343.3–845.5	0.005	712.5	543.3–868.8	
Second	541.0	242.3–842.8 324.0–810.0		468.0	245.0–655.0		506.0	355.0–769.0	0.0002
Adherence									
Good	567.0	247.0–848.0	0.869	559.0	361.0–814.5	0.159	601.0	433.0–810.0	
Poor	554.0	300.0–814.3		462.0	286.5–705.0		680.5	418.0–863.0	0.674
Clinical stage									
Failure	153.5	22.8–601.8	0.057	298.0	133.3–531.5	0.013	529.0	382.3–820.5	
Success	567.0	265.5–848.5		545.0	350.0–797.0		644.0	431.5–817.5	0.264
PVL ranges									
UND	724.5	518.0–927.3	-	626.0	438.8–919.0	-	699.0	454.3–854.3	-
VS	573.0	413.8–693.5		575.5	379.0–703.3		628.0	532.0–801.0	
VF	261.0	140.0–522.0		389.0	195.0–635.0		526.0	355.0–735.8	

**Table 3:** Results of two-way ANOVA analysis.


Degree of freedom	Sum of square	Mean sum of square	*F* value	*p*-value
2	881,960	440,980	3.9	0.021
692	78,244,396	113,070		

### Immunological response and sociodemographic characteristics

**Figure 3:**
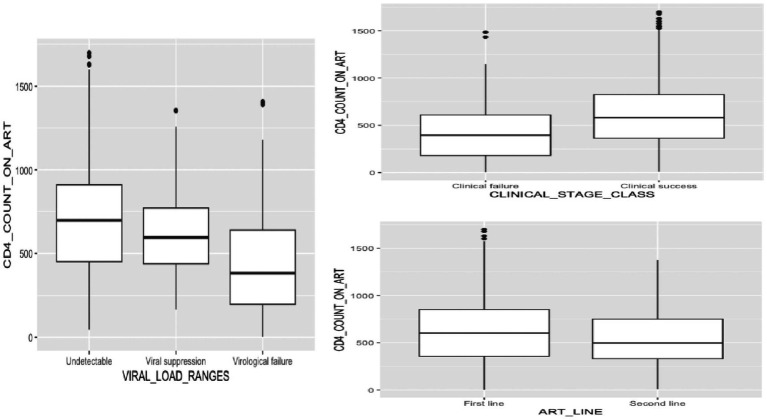
CD4+ count dynamics in adolescents living with HIV: effect of clinical, virological, and antiretroviral treatment regimen characteristics.

Furthermore, statistical analysis comparing CD4+ counts between participants on first-line ART and those on second-line ART revealed notable differences across the study’s phases. At enrolment, the comparison yielded a non-significant *p*-value of 0.876, suggesting no substantial distinction in CD4+ counts between the two treatment groups. However, as the study progressed into 6-month and 12-month follow-ups, the *p*-values decreased significantly to 0.005 and 0.0002, respectively (Figure 3; Table 2). Investigating CD4+ counts between early and late adolescence cohorts revealed significant differences across study phases. At enrolment, a low *p*-value (*p* < 0.0001) indicated significant variation, with early-age adolescents having higher median CD4+ counts compared to older adolescents. Similar trends persisted at 6-month (*p* = 0.0005) and 12-month (*p* < 0.0001) follow-up, underlining persistent disparities between early and late adolescence groups (Figure 2; Table 2). Moreover, participants with good adherence to ART exhibited higher median CD4 counts compared to those with poor adherence (Figure 2). However, these differences were non-significant across all three phases of the study (Table 2).

### Immunological response according to clinical status and virological response

Comparing CD4+ count among ALHIV classified based on their clinical status revealed varying levels of significance (Figure 3). In phase 1, a potential difference in CD4 count between ALHIV classified as clinically successful versus those classified as clinically failed was suggested, however, this analysis had only a borderline significance (*p* = 0.0572). Phase 2 yielded a more significant result, with a *p*-value of 0.0125, but in phase 3, the comparison showed non-significant results, with a *p*-value of 0.2637, suggesting no significant difference in CD4+ count based on clinical outcomes success (Table 2). Finally, assessing the impact of plasma viral load (PVL) ranges on CD4+ count demonstrated a statistically significant effect [*F*(2, 692) = 58.89, *p* < 0.001] (Table 4). Further assessment across the three levels of PVL (viral suppression, virological failure, and undetectable viremia) revealed statistically significant variations in CD4+ count across these groups (Figure 3). The *p*-values obtained from the *post hoc* test were 0.0119, <0.001, and < 0.001, corresponding to the pairwise comparisons between viral suppression and undetectable viremia, virological failure and undetectable viremia, and virological failure and viral suppression, respectively (Table 5).

**Table 4:** Results of repeated measures two-way ANOVA analysis.

	Degree of freedom	Sum of square	Mean sum of square	F value	*p*-value
PVL ranges	2	11,509,230	5,754,615	58.89	< 0.001
Residuals	692	67,617,126	97,713		

**Table 5:** Results of Tukey HSD tests.

Pairs	Difference	95% Confidence Interval	*p*-value
VS – Undetectable	−97.56	−177.37 to −17.74	0.0119
VF – Undetectable	−282.77	−343.92 to −221.62	<0.001
VF – VS	−185.21	−267.95–102.48	<0.001

### Correlation of CD4 count with age and ART duration

**Figure 4:**
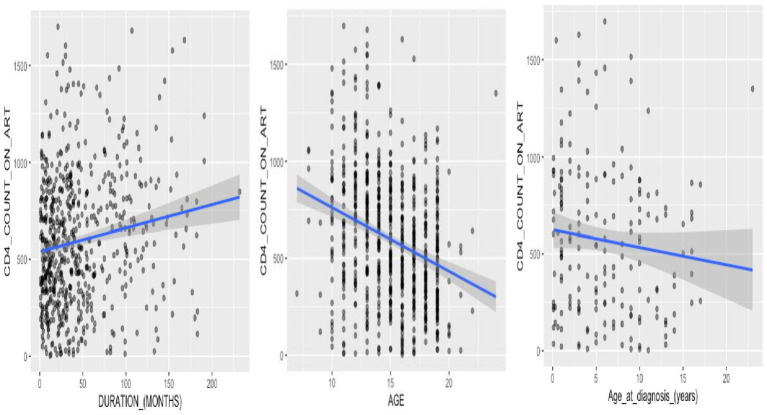
Correlation analysis of CD4+ count dynamics in adolescent HIV patients: assessing associations with age at HIV diagnosis, age, and duration of treatment.

Assessing the relationship between age and CD4+ count among ALHIV revealed that there was a statistically significant moderate negative correlation between absolute CD4+ count on antiretroviral therapy and adolescence age (correlation coefficient, *r* = −0.28, confidence interval = −0.35 to −0.21, *p*-value <0.0001) (Figure 4). Conversely, there was a statistically significant weak positive correlation between CD4+ count on ART and duration of treatment (*r* = 0.15, CI = 0.07–0.23, *p* = 0.0001) (Figure 4). A correlation test between CD4+ count and age at HIV diagnosis yielded a weak negative correlation between CD4+ count on antiretroviral therapy and increasing age at HIV diagnosis, but this correlation was not statistically significant (*r* = −0.11, CI = −0.25 – 0.04, *p* = 0.143) (Figure 4).

### Multivariate analysis

The results of the final multivariable linear regression model with and without study site (as random effect) were presented in Table 6. Details about the model selection were presented in the Supplementary Table S1. Supplementary Figure S1 illustrates the validation of model assumptions, confirming linearity, normality, homoscedasticity, and the absence of outliers through graphical examination. A linear regression model was applied to analyze the relationship between CD4+ count on ART and various predictors including site group, gender, age ranges, educational level, age at diagnosis, ART line, duration on treatment, self-reported adherence, clinical stage class, viral load ranges, and the interaction between sex and educational level (Table 6). Among the predictors, males exhibited a decrease in CD4+ count on ART compared to females [Estimate: −198.07 (95% CI: −376.81 to −19.32), *p* < 0.001]. Similarly, participants in the late adolescence age range had lower CD4+ counts compared to those in early adolescence [−185.93 (−306.30 to −65.56), *p* = 0.003]. Notably, participants with virological failure had significantly lower CD4+ counts compared to those with undetectable viral loads [−354.21 (−466.96–241.47), *p* < 0.0001] (Table 6). Moreover, the interaction between gender and educational level showed marginal significance, suggesting that there may be some interaction effect between being male and having a secondary educational level [206.29 (−0.01–0.01), *p* = 0.062]. However, this interaction effect was not statistically significant (Table 6).

**Table 6:** Multivariate linear regression for CD4+ count with and without study site as random effect.

	Linear regression		Linear mixed-effect regression	
	Estimates (95% CI)	*p*-value	Estimates (95% CI)	*p-value*
Intercept	739.71 (459.46–1019.95)	**<0.001**	807.66 (529.78–1085.54)	**<0.001**
Gender				
Female	ref			
Male	−198.07 (−376.81 to −19.32)	**0.030**	−200.63 (−379.32 to −21.95)	**0.028**
Age ranges				
Early adolescence	ref		ref	
Late adolescence	−185.93 (−306.30 to −65.56)	**0.003**	−181.08 (−301.08 to −61.09)	**0.003**
Educational level				
Secondary	−160.67 (−325.93–4.58)	0.057	−158.93 (−324.16–6.29)	0.059
Age at diagnosis	−4.19 (−16.62–8.24)	0.506	−5.04 (−17.36–7.28)	0.420
ART line				
First	ref			
Second	69.57 (−95.06–234.19)	0.405	78.81 (−84.81–242.43)	0.343
Duration on treatment	1.03 (−0.26–2.31)	0.117	1.08 (−0.21–2.36)	0.100
Self-reported adherence				
Good	ref			
Poor	26.58 (−0.24 to −0.10)	0.641	23.18 (−88.84–135.19)	0.683
Clinical stage class				
Failure	ref			
Success	75.75 (−0.05–0.13)	0.469	74.98 (−131.47–281.42)	0.474
Viral load ranges				
Undetectable	ref		ref	
Viral suppression	−100.67 (−292.59–91.26)	0.302	−99.26 (−291.18–92.65)	0.309
Virological failure	−354.21 (−466.96–241.47)	**<0.0001**	−353.08 (−465.81 to −240.36)	**<0.001**
Interaction				
Gender [Male]* Educational level [Secondary]	206.29 (−0.01–0.01)	0.062	209.78 (−6.94–426.51)	0.058
Random effects				
σ^2^_res_			110163.12	
τ_00 SITE_GROUP_			4277.77	
ICC			0.04	
N_Site_group_			2	
Observations			170	
Marginal R^2^/Conditional R^2^			0.318 / 0.343	

σ^2^_res_, variability within hospitals. τ_00 SITE_GROUP l_, variability between hospitals. ICC, Intraclass correlation coefficient = σ^2^_Site_group_/(σ^2^_res_ + σ^2^_Site_group_). In bold are the significant *p*-values.

The linear regression model was extended to a linear mixed-effects regression model with random intercepts specified for each participant’s site group to account for variability between study sites (Table 6). The summary of the model revealed several significant fixed effects. Being male was associated with a decrease in CD4+ count compared to females, [−200.63 (−379.32 to −21.95), *p* = 0.028]. Similarly, individuals in the late adolescence age range [−181.08 (−301.08 to −61.09), *p* = 0.003] and those with virological failure [−353.08 (−465.81 to −240.36), *p* < 0.001] exhibited lower CD4+ counts compared to their respective reference categories. Furthermore, the interaction between gender and educational level showed a marginally significant effect, suggesting that the association between male gender and CD4+ count may vary depending on whether individuals have attained a secondary education level [209.78 (−6.94–426.51), *p* = 0.058] (Table 6).

Overall, the model demonstrated moderate explanatory power, with a marginal R-squared value of 0.318, indicating that approximately 31.8% of the variance in CD4+ count on ART was explained by the predictors included in the model (Table 6). The conditional R-squared value was 0.343 (34.3%), suggesting that the model’s explanatory power remains robust after adjusting for the number of predictors. The F-statistic was highly significant (*p* < 0.001), indicating that the overall model was statistically significant in predicting CD4+ count on ART. In addition, the random effects analysis revealed significant variability between study sites, with a variance of 4277.77. This underscored the importance of accounting for site-level differences when analyzing CD4+ count data in this context.

## Discussion

Our study identified important factors that influence CD4+ counts among perinatally-infected adolescents living with HIV (ALHIV) and the importance of considering demographic, clinical, and contextual factors in HIV care and management. Our findings suggest that tailored interventions addressing gender disparities, age at diagnosis, educational attainment, and healthcare delivery systems are crucial for optimizing health outcomes and improving the well-being of ALHIV.

Most of the study participants hailed from urban areas, possibly linked to improved access to healthcare services, including ART clinics and support systems, compared to their counterparts in rural areas. This observation aligns with findings from previous studies conducted in Cameroon (25, 26) but contrasts with those reported in Kenya (27). Urban settings typically have more developed healthcare infrastructure and resources, which may contribute to higher retention rates among ALHIV (27). However, the shifting demographic trends observed in our study, with an increasing proportion of older adolescents’ overtime, suggest evolving dynamics that warrant careful consideration. Older adolescents may encounter distinct challenges related to HIV management, treatment adherence, or psychosocial support needs compared to their younger counterparts (28, 29). Understanding of these age-specific nuances is essential for tailoring interventions and support ART services effectively to meet the diverse needs of ALHIV across different age groups (30) a finding similar to other studies in Cameroon (27, 31).

Furthermore, our results reveal consistent patterns in the participants’ demographic and educational characteristics over time. The median age of five years at HIV diagnosis among perinatally infected adolescents raises concerns about missed opportunities for early treatment, underscoring the importance of enhancing HIV testing and prevention efforts, particularly among at-risk populations like perinatally infected adolescents as seen in other countries (27, 32). Moreover, the greater representation of female participants compared to males may indicate gender-specific disparities in healthcare-seeking behaviors or HIV prevalence among ALHIV, consistent with national data (33–36). This contrasts with the predominance of males reported in a European study (37). The predominance of participants at the secondary educational level, and the marginally significant interaction between male gender and attainment of this level of education regarding CD4+ count suggest that educational attainment may influence healthcare access, ART adherence, or other factors affecting immunological responses, which may vary between males and females. Further research is needed to fully understand this association.

In terms of clinical outcomes, most participants achieved clinical success, with the majority on first-line RTI-based ART regimens and reporting good adherence. However, this raises concerns about prolonged exposure to suboptimal regimens with low barriers to drug resistance, a significant consideration in HIV treatment (38–40). Consistent with findings from similar settings (31, 38, 41). Moreover, the fluctuations observed in CD4+ counts suggest dynamic changes in immune response and treatment effectiveness over the follow-up periods. Analysis of the ART regimens administered revealed a variety of first-and second-line treatment combinations, consistent with findings from similar settings (39, 42). This diversity emphasizes the significance of a personalized therapy approach tailored to factors like HIV drug resistance, side effects, and patient response, all crucial for enhancing treatment outcomes. These findings highlight the dynamic nature of HIV care, emphasizing the importance of continuous monitoring of CD4+ cell counts, viral loads, and treatment plans to ensure optimal clinical outcomes for ALHIV receiving ART (43, 44).

Analyzing median CD4+ counts across different study population characteristics revealed several key findings. The absence of a significant difference in the median CD4+ counts between males and females suggests that gender may not have a significant impact on CD4+ fluctuations over time (45), contrasting with studies indicating women’s susceptibility to immunological failure and virological failure (45–49). Furthermore, the lack of significant disparity in CD4+ counts between urban and rural study sites implies that location may not influence CD4+ counts among ALHIV, potentially due to effective adherence counseling provided at both sites. Unlike findings reported in Zimbabwe where CD4+ counts were higher in urban settings (50).

The variation in median CD4+ counts across educational levels, with the highest values observed among participants in primary education, followed by secondary education, and the lowest among those with university education, prompts intriguing considerations. One plausible explanation is that adolescents at the primary education level may benefit from enhanced parental/guardian oversight, contributing to better adherence and health management. Meanwhile, older adolescents pursuing higher education may encounter heightened social stigma and challenges associated with disclosing their HIV status, potentially impacting their psychological well-being and treatment adherence (51, 52).

Investigating CD4+ counts between early and late adolescence cohorts demonstrated a statistically significant difference overtime (40, 53–55). This could be due to physiological changes, variations in treatment adherence, healthcare access, and psychosocial factors like stress, stigma, and mental health issues. Therefore, it is important to consider age-related factors when assessing immune health and treatment response among ALHIV to identify and tackle the unique needs and challenges faced by different adolescent age groups.

Our study findings suggested that plasma viral load (PVL) levels had a significant impact on CD4+ counts among ALHIV. Participants with viral suppression had different CD4+ counts compared to those with undetectable viremia or virological failure. The pairwise comparisons further emphasized these differences, indicating that maintaining viral suppression is crucial for preserving immune health and optimizing treatment outcomes. Effective management of PVL through ART is essential to achieve and sustain viral suppression, which in turn helps to maintain or improve CD4+ counts. Therefore, strategies aimed at achieving and maintaining viral suppression should be prioritized in HIV treatment and care protocols to ensure better health outcomes for ALHIV (13, 56–58).

Further analysis revealed several significant fixed effects on immune responses. Being male was associated with a decrease in CD4+ count compared to females which aligns with studies in other African countries (45, 59, 60). This could be attributed to females generally mounting stronger immune responses to infections, including HIV, as well as potential differences in healthcare-seeking behavior or treatment adherence (61, 62). Similarly, the lower CD4+ counts among ALHIV in the late adolescence age range could be attributed to various factors. These include a longer duration of HIV infection leading to more advanced disease progression (63, 64), increased social and emotional stress, and potential decline in treatment adherence as adolescents transition into adulthood. Additionally, ALHIV experiencing virological failure exhibited lower CD4+ counts which may be linked to poor ART adherence, drug resistance, suboptimal treatment regimens, or challenges in accessing healthcare services (33, 38, 65).

More still, the interaction between gender and educational level showed a marginally significant effect, suggesting that the relationship between male gender and CD4+ count may vary depending on whether individuals have attained a secondary education level. This could be due to socioeconomic factors associated with education, such as access to healthcare services, or lifestyle factors. However, further research is needed to fully understand the underlying mechanisms driving this interaction (37).

The results highlight the importance of prioritizing close immune monitoring in the perinatal ALHIV population, advocating for frequent CD4+ count tests to optimize strategies for rapid immune recovery, as recommended by WHO in 2017 (66, 67). While viral load (VL) testing is considered the gold standard for monitoring treatment response, it is crucial for countries to maintain their CD4+ cell count testing capacity, alongside efforts to provide quality VL testing to support comprehensive and effective HIV care and management (8).

In our longitudinal study, missing laboratory data and participant loss to follow-up presented challenges, as methods like multiple imputation and sensitivity analysis were not used to evaluate the impact of missing data. This limitation may have affected data quality and the applicability of findings to the target population. However, the study’s strengths included a multi-time point follow-up, personalized monitoring, and repeated assessments, ensuring robust within-program comparisons of immunovirological responses and minimizing potential confounders. Despite these strengths, relying on complete case analysis may have introduced bias, highlighting the need for more advanced methods to handle missing data.

## Conclusion

In a nutshell, these findings emphasize the importance of prioritizing close immune monitoring for ALHIV, particularly those in the older adolescent age category, of male gender, and those experiencing virological failure. It is imperative that the care strategy, as recommended by WHO, for such cases includes CD4+ count assessment, given its significance as the most relevant immunological parameter correlated with virological response in this population.

## Data Availability

The raw data supporting the conclusions of this article will be made available by the authors, without undue reservation.

## Footnotes

^1^http://circb.cm/btc_circb/web/
