# Supplementary material for: Empowering adolescents living with perinatally-acquired HIV: tailored CD4+ count assessment for optimized care, the EDCTP READY-study
Source: Front Med (Lausanne). 2024 Sep 20;11:1457501. doi: 10.3389/fmed.2024.1457501 (PMC11450861; doi:10.3389/fmed.2024.1457501)
Supplement: Supplementary file 1 [file Data_Sheet_1.docx]

**Supplementary Materials**

**Table S1**: Model selection

| **Model index** | **Predictors** | **R^2^** | **Adjusted R^2^** | **AIC** |
| --- | --- | --- | --- | --- |
| 1 | Site group | 0.0024 | 0.0009 | 10068.296 |
| 2 | Gender | <0.0001 | -0.0014 | 10069.945 |
| 3 | Age ranges | 0.0838 | 0.0824 | 10009.161 |
| 4 | Educational level | 0.0222 | 0.0179 | 10058.362 |
| 5 | Age at diagnosis | 0.0119 | 0.0064 | 2695.962 |
| 6 | ART line | 0.0120 | 0.0106 | 10061.559 |
| 7 | Duration on treatment | 0.0231 | 0.0215 | 9036.746 |
| 8 | Adherence | 0.0007 | -0.0008 | 10069.507 |
| 9 | Clinical stage class | 0.0184 | 0.0171 | 10057.062 |
| 10 | Viral load ranges | 0.1455 | 0.143 | 9962.719 |
| 11 | Site group + Gender | 0.0024 | -0.0005 | 10070.290 |
| 12 | Site group + Gender + Age ranges | 0.0905 | 0.0866 | 10008.002 |
| 13 | Site group + Gender + Age ranges + Educational level | 0.0942 | 0.0863 | 10011.240 |
| 14 | Site group + Gender + Age ranges + Educational level + Age at diagnosis | 0.1665 | 0.133 | 2676.985 |
| 15 | Site group + Gender + Age ranges + Educational level + Age at diagnosis + ART line | 0.1689 | 0.1304 | 2678.479 |
| 16 | Site group + Gender + Age ranges + Educational level + Age at diagnosis + ART line + Duration on treatment | 0.1627 | 0.1265 | 2503.249 |
| 17 | Site group + Gender + Age ranges + Educational level + Age at diagnosis + ART line + Duration on treatment + Adherence | 0.1634 | 0.1218 | 2505.104 |
| 18 | Site group + Gender + Age ranges + Educational level + Age at diagnosis + ART line + Duration on treatment + Adherence + Clinical stage class | 0.1765 | 0.1302 | 2504.420 |
| 19 | Site group + Gender + Age ranges + Educational level + Age at diagnosis + ART line + Duration on treatment + Adherence + Clinical stage class + Viral load ranges | 0.334 | 0.2876 | 2472.349 |
| 20 | Site group + Gender + Age ranges + Educational level + Age at diagnosis + ART line + Duration on treatment + Adherence + Clinical stage class + Viral load ranges + Site group*Educational level | 0.3341 | 0.2832 | 2474.324 |
| 21 | Site group + Gender + Age ranges + Educational level + Age at diagnosis + ART line + Duration on treatment + Adherence + Clinical stage class + Viral load ranges + Site group*Age at diagnosis | 0.3343 | 0.2835 | 2474.252 |
| 22 | Site group + Gender + Age ranges + Educational level + Age at diagnosis + ART line + Duration on treatment + Adherence + Clinical stage class + Viral load ranges + Site group*Adherence | 0.3355 | 0.2847 | 2473.959 |
| 23 | Site group + Gender + Age ranges + Educational level + Age at diagnosis + ART line + Duration on treatment + Adherence + Clinical stage class + Viral load ranges + Gender*Educational level | 0.3486 | 0.2988 | 2470.567 |
| 24 | Site group + Gender + Age ranges + Educational level + Age at diagnosis + ART line + Duration on treatment + Adherence + Clinical stage class + Viral load ranges + Age ranges*Educational level | 0.3381 | 0.2875 | 2473.283 |
| 25 | Site group + Gender + Age ranges + Educational level + Age at diagnosis + ART line + Duration on treatment + Adherence + Clinical stage class + Viral load ranges + Age ranges*ART line | 0.3362 | 0.2854 | 2473.788 |
| 26 | Site group + Gender + Age ranges + Educational level + Age at diagnosis + ART line + Duration on treatment + Adherence + Clinical stage class + Viral load ranges + Age ranges*Duration on treatment | 0.340 | 0.2895 | 2472.811 |
| 27 | Site group + Gender + Age ranges + Educational level + Age at diagnosis + ART line + Duration on treatment + Adherence + Clinical stage class + Viral load ranges + Age ranges*Adherence | 0.334 | 0.2831 | 2474.341 |
| 28 | Site group + Gender + Age ranges + Educational level + Age at diagnosis + ART line + Duration on treatment + Adherence + Clinical stage class + Viral load ranges + Educational level*Duration on treatment | 0.3371 | 0.2864 | 2473.552 |
| 29 | Site group + Gender + Age ranges + Educational level + Age at diagnosis + ART line + Duration on treatment + Adherence + Clinical stage class + Viral load ranges + Educational level*Adherence | 0.3369 | 0.2863 | 2473.585 |
| 30 | Site group + Gender + Age ranges + Educational level + Age at diagnosis + ART line + Duration on treatment + Adherence + Clinical stage class + Viral load ranges + Age at diagnosis*Duration on treatment | 0.3349 | 0.284 | 2474.120 |
| 31 | Site group + Gender + Age ranges + Educational level + Age at diagnosis + ART line + Duration on treatment + Adherence + Clinical stage class + Viral load ranges + Age at diagnosis*Adherence | 0.334 | 0.2831 | 2474.331 |
| 32 | Site group + Gender + Age ranges + Educational level + Age at diagnosis + ART line + Duration on treatment + Adherence + Clinical stage class + Viral load ranges + Age at diagnosis*Clinical stage class | 0.3358 | 0.285 | 2473.889 |
| 33 | Site group + Gender + Age ranges + Educational level + Age at diagnosis + ART line + Duration on treatment + Adherence + Clinical stage class + Viral load ranges + Age at diagnosis*Viral load ranges | 0.3368 | 0.2815 | 2475.630 |

**
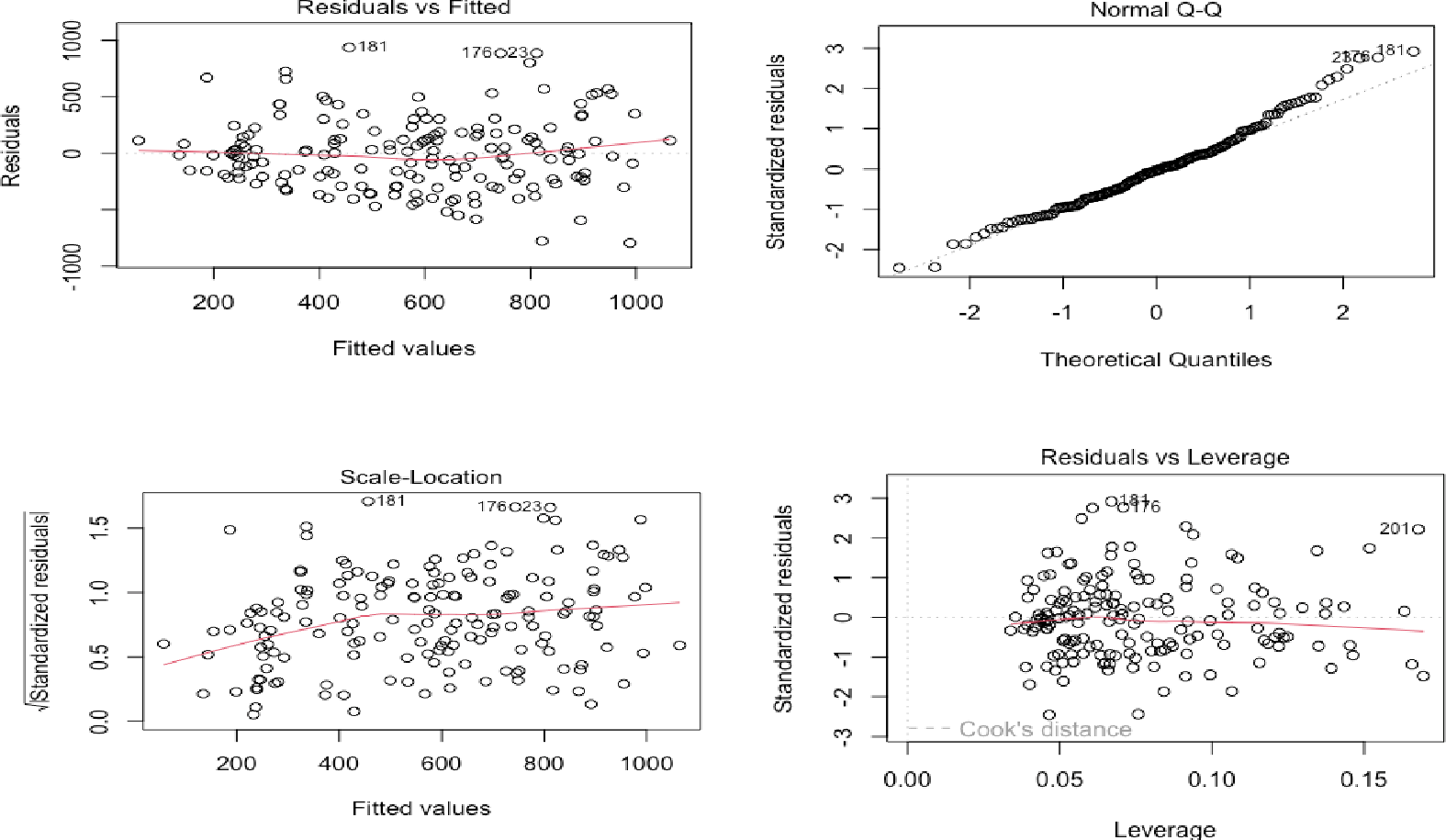
**

**Figure S1**: Diagnostic plots for multivariate linear regression
